# Improving Implementation of an Electronic Prescription System for COVID-19 Vaccination in the Czech Republic: Process Modeling Approach

**DOI:** 10.2196/41575

**Published:** 2023-03-03

**Authors:** Jiri Berger, Jan Bruthans, Jiří Kofránek

**Affiliations:** 1 Institute of Pathological Physiology First Faculty of Medicine Charles University in Prague Praha Czech Republic; 2 Department of Biomedical Technology Faculty of Biomedical Engineering Czech Technical University in Prague Kladno Czech Republic; 3 Department of Anesthesiology and Intensive Care General Teaching Hospital Praha Czech Republic

**Keywords:** eHealth, electronic prescription, process modeling, state diagram, COVID-19, vaccination, vaccine, medical, communication, platform

## Abstract

**Background:**

It is very difficult to find a consensus that will be accepted by most players when creating health care legislation. The Czech electronic prescription system was launched in 2011 and new functions were introduced in 2018. To ensure that these functions will not conflict with any other existing law, a process modeling tool based on the patent “Method and system for automated requirements modeling” was used successfully in the Czech Republic for the first time.

**Objective:**

The aim of this project was to develop another successful application of process modeling to add COVID-19 vaccination records to the existing electronic prescription system.

**Methods:**

The method employed was based on the mathematical theory of hierarchical state diagrams and process models. In the first step, sketches that record the results of informal discussions, interviews, meetings, and workshops were prepared. Subsequently, the architecture containing the main participants and their high-level interactions was drafted. Finally, detailed process diagrams were drawn. Each semiresult was discussed with all involved team members and stakeholders to incorporate all comments. By repeating this procedure, individual topics were gradually resolved and the areas of discussion were narrowed down until reaching complete agreement.

**Results:**

This method proved to be faster, clearer, and significantly simpler than other methods. Owing to the use of graphic tools and symbols, the risk of errors, inaccuracies, and misunderstandings was significantly reduced. The outcome was used as an annex to the bill in the legislative process. One of the main benefits of this approach is gaining a higher level of understanding for all parties involved (ie, legislators, the medical community, patient organizations, and information technology professionals). The process architecture model in a form of a graphic scheme has proven to be a valuable communication platform and facilitated negotiation between stakeholders. Moreover, this model helped to avoid several inconsistencies that appeared during workshops and discussions. Our method worked successfully even when participants were from different knowledge areas.

**Conclusions:**

The vaccination record process model was drafted in 3 weeks and it took a total of 2 months to pass the bill. In comparison, the initial introduction of the electronic prescription system using conventional legislative methods took over 1 year, involving immediate creation of a text with legislative intent, followed by paragraph-by-section wording of the legislation that was commented on directly. These steps are repeated over and over, as any change in any part of the text has to be checked and rechecked within the entire document. Compared with conventional methods, we have shown that using our method for the process of modification of legislation related to such a complex issue as the integration of COVID-19 vaccination into an electronic prescription model significantly simplifies the preparation of a legislative standard.

## Introduction

Health care legislation has various specificities. Several different interest and influence groups (including insurance companies, patient associations, medical chambers, and others) bring their influences and requirements into this already complex issue and it is very difficult to find a consensus that will be accepted by most players. Previous experiences with the common legislation processes in the Czech Republic show that it typically takes 1 year or longer for a motion to be accepted. Usually, there are several back-and-forth cycles and in some cases the whole process might even be stopped for a while.

Relationships between influence groups and their information requirements must be described in a sufficiently transparent manner for future successful implementation of health care legislation. This description forms the basis for the design of the architecture and functions of information systems. Such description must be comprehensible to all parties, regardless of whether they have computer, economic, legislative, or scientific (including medical) knowledge. Moreover, defining functions of the system via the above-mentioned description can be used for creating related legislation, which is necessary due to the nature of health care as a public service.

The first attempts at legislation enabling electronic prescriptions (EPs) in the Czech Republic date back to 2006. Since 2009, the State Institute for Drug Control (SÚKL) has been authorized by law to develop and administer the electronic prescription system (EPS). Such a system had only a few basic rules established by law. The Czech EPS (named eRecept) was launched more than 2 years later in May 2011. At that time, the system architecture consisted of a central server with storage and a defined communication interface. Physicians issuing an EP or pharmacists dispensing medicines based on an EP needed an end-user system. Users were expected to obtain these systems from commercial vendors, which caused a very slow uptake of EPS in everyday life, as it was used on a voluntarily basis at that time. The only functionality of the EPS was storing the prescription and issuing an identifier, which was printed in a barcode on the prescription. This identifier was subsequently scanned at the pharmacy providing data from a central repository. While this implementation is consistent with the basic definition of an EPS [1], it completely lacks more advanced features such as lists of dispensed drugs or even checking for adverse drug interactions. A qualified electronic signature was required to authenticate each user, which was another barrier to implementation.

For several years, only a very low percentage of prescriptions were issued using this EPS with most prescriptions remaining paper-based. In addition, only a limited number of physicians used the EPS at all. This was probably due to the lack of advanced features, as most users had no reason to use the EPS. Usage of the EPS was made mandatory in January 2018. Although this was initially not well-received by professionals and the lay public, obligatory use has significantly increased uptake of the system; the share of EPs among all prescriptions issued increased from 2% in 2016 to over 80% in 2018, and similarly, the share of physicians issuing prescriptions has increased from around 5% in 2016 to over 84% in 2018 [2]. New functions of the EPS were introduced such as sending prescription identifiers by email or SMS text messaging. Patients could also access the list of medications prescribed to them through a simple web application or mobile (tablet/phone) app.

Many other EPSs developed in the European Union at that time also enabled physicians and pharmacists to view the patient medication list; however, this function was not available in the Czech EPS in 2018 [3]. To introduce the EPS, a new bill had to be drafted, which required solving some complex issues, including detailed mechanisms for data sharing and methods to restrict and control professionals’ access to data. To ensure that the EPS and its new functionalities would not conflict with any other existing law, a process modeling tool based on the patent “Method and system for automated requirements modeling” [4] was used successfully in the Czech Republic for the first time. The final process model became a part of the accepted bill. Based on this bill, a medication list accessible to all entitled professionals was launched in June 2020.

With occurrence of the COVID-19 pandemic, new challenges emerged, and a national vaccination record (VR) was needed. The decision was made to use and enhance the existing EPS and its patient medication list with this new VR functionality rather than constructing an entirely new system. We here introduce an innovative process modeling method that was developed to accelerate the process and ensure its success.

## Methods

### Process Model as a Tool for Discussion

Different methods of process modeling in health care have been traditionally used along with the general development of process models in the information technologies area. One of the earliest and most widely used methods is the Unified Modeling Language (UML) to visualize existing situations in health care. The most relevant health care area where process models have long been utilized is the well-known international standard Health Level 7 (HL7) as an exchange format between different software applications across health care. Moreover, there are several references to using the Business Process Model and Notation (BPMN) method as part of graphical notation across complex health care processes. Journey maps is another process visualization method that is used in a similar manner to BPMN. The specifics of health care are based on very complex problems that have to be modeled. For example, complex causal process diagrams [5] have been used for specifying the causal linkages in complex systems, organizing interdisciplinary research, and serving as a basis for modeling. Similarly, five business process modeling techniques (data flow diagram, flow chart, integrated computer-aided manufacturing definition for function modeling, lean value stream map, and the patient journey modeling tool) have been used to represent selected patient journey models to obtain the necessary background for quantitative and qualitative analyses [6].

Among the various available methods, we decided to use hierarchical state machines–based process modeling because of its pure mathematical foundation as well as easy-to-understand visualization based on animated process diagrams.

State diagrams and process models were used as a type of imaginary “pencil” to draw the process architecture of the EPS/VR, which was then used as a communication tool for interdisciplinary understanding.

The first step focuses on an informal discussion of the problem. This is then gradually converted into a formally and factually correct solution of processes and the design of an information system that supports the identified processes. For this purpose, there are usually several discussions and workshops to improve the diagram for the next version. The following modeling phases were used to work on the EPS/VR model: (1) sketches, (2) architecture, and (3) process diagrams.

### Process Modeling Phases

#### Sketches

When modeling complex systems, as much information as possible should be recorded at the very beginning. It is therefore not possible to be overly bound by formalism, as important messages could be omitted when a strict methodology is followed. Sketches are used for the initial capture of information, as it is not yet possible at this stage to strictly model according to the types of objects and the rules of the methodology.

At this stage, the initial “model” is created only on paper, resulting in an almost nonreproducible doodle. However, many years of experience show that going through the “scribbling, deleting, throwing” phase is necessary for finding the “right” elements of the ultimate real model, as the understanding of the entire issue and context emerges only over time.

Already at this phase, the main procedural participants have to be identified and confirmed: the patient, physician, pharmacist, EPS/VR, and necessary procedural part of the consent management. At the same time, the main states of the process participants have to be defined. For example, the patient might be “receiving information about possible revaccination planning” or “receiving a vaccination report,” whether the report be delivered electronically or on paper. Therefore, the state of the vaccination report might be “created in a paper form” or “created in an electronic version.”

In the first phase of modeling, close and functional cooperation with future users and regulatory authorities is necessary. It is impossible to succeed without detailed knowledge of all possible conditions and legislative requirements for prescribing drugs in the Czech Republic. This knowledge is generally not available to information systems architects.

In this sketches phase, one should not be entrenched in debates on the topic of activities (ie, who does what), as these debates are ultimately unproductive at this early stage. However, it can be important to trace who gets into which state, such as the state examples given above (ie, “receiving information about possible revaccination planning” or “receiving a vaccination report”).

#### Architecture

The next step is to check the possibility of combining the defined states of individual participants together into one or more “stories.” This enables individual events that are taking place in the system to be described by the elements of the created model. This is when the formal phase of architecture begins. In other words, the architecture is no longer a sketch but rather an assembled diagram.

The architecture formally displays: (1) the process participants (eg, patient, nurse, physician, consent management), (2) scenario groups consisting of simple stories (eg, obtaining a recipe, dispensing medicine), (3) scenarios/stories, and (4) links between scenarios (arrows).

#### Process Diagrams

**Figure 1:**
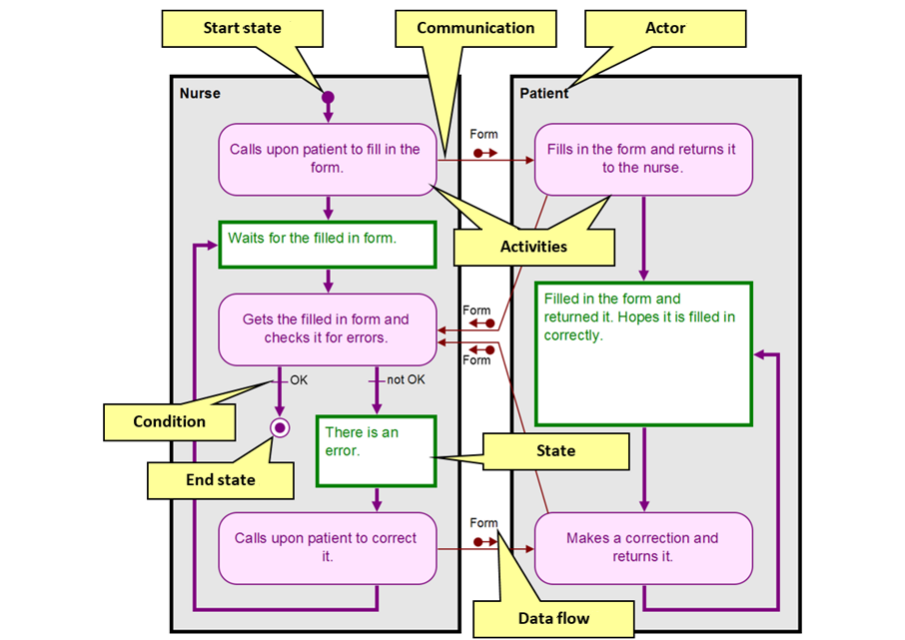
Example process diagram for hospital registration between a nurse and patient.

An example of a process diagram showing the registration process in a hospital’s reception is provided in Figure 1. The two players that participate in this process are the nurse and the patient. The process consists of passing a form to the patient, filling in the form, handing the filled-in form back to the nurse, checking if the form is filled in correctly, and if not, returning the form to the patient to correct. The process ends when the nurse finds no errors in the form.

In the method terminology, the players who act in a process are called *actors*. Each actor performs certain activities and then enters into a specific state. Actors communicate with each other through *communication*. The communication can carry a data flow. When there is more than one possibility on how to continue the process, more arrows are leaving an activity or state, and each of them has an associated condition. In the example provided in Figure 1, there are two conditions: “OK” and “not OK.”

An actor (large grey rectangles in Figure 1) is a person or thing that performs actions. The actor can also be referred to as a *player* or a *role*. Actors should not be called by their names (eg, Mr Scott) but rather by their occupation (eg, nurse) or role in the particular process (eg, applicant, customer, patient). Machines or software systems can also be actors.

An activity (pink rounded rectangles in Figure 1) is a small but compact piece of action performed by an actor in a given process. The activity should not be too small (eg, the pressing of a key) but should also not be too large (ie, the whole process).

A state (green-white rectangles in Figure 1) describes a situation that an actor is in after performing a particular activity. Note that the activities and states alternate. A state is often described by a passive sentence. We consider the state to be more important than the activity; that is, the activity is simply a way to move from one state to the other. However, the most important activities can be highlighted in the diagram.

Start and end states (violet or double circles in Figure 1) represent special types of states. In the start state, the entire process is initiated, whereas the process ends in the end state. The process must start with at least one start state and it often ends with more than one result, leading to multiple end states. Either one actor is connected to the start and end states, or every (or nearly every) actor is connected to the start state and several of the end states.

Communication (thin arrows between two activities in Figure 1) represents any type of interaction occurring between two actors. Communication is an active act, and therefore represents a link only between two activities, each of them executed by a different actor. Communication starts with the activity of the actor who initiates the interaction (eg, questioner, incomer) and points to the activity of the actor who will be affected by this communication. Communication can often bear data flows (small arrows with a label). Data flows can flow either in the direction of the communication (eg, question, call) or in the opposite direction (eg, answer). Data flows are sometimes omitted, as they are usually used only for emphasizing certain important data or documents.

Finally, a condition (line or arrow overstrike with a label in Figure 1) is a point where the process splits into several branches. Each branch is specified by a condition. The condition does not take the form of a question (eg, “Did the accused appear before a court?”) but rather the form of the answer to that question (eg, “The accused appeared before a court” or “The accused did not appear before a court”). The semantics of the condition are as follows: if the condition is true, the process moves along that line or arrow; if the condition is not true, the process does not move along that line or arrow. Conditions are mostly used on transitions (thick violet line or arrow connecting an activity with a state), but can also be used in communications in certain cases.

In the textual descriptions of the two examples above, most of the initial features of crafting diagrams have to be introduced. The method then continues by reinforcing our understanding of these basic components by considering several further examples.

In the process model, each scenario, in addition to the name, is also characterized by the three types of descriptive data: (1) initiation, describing the status of the process participant in which the scenario is activated; (2) actions, describing the actions that the scenario performs (eg, the pharmacist converts the paper prescription to electronic form and further treats it as an electronic form); and (3) result, describing the status of the process participant after completion of the scenario.

The links between the scenarios show how the individual scenarios performed by the process participants follow each other.

### Ethical Approval

A joint Institutional Review Board of First Medical Faculty and General Teaching Hospital consented to the publication of our research (209/23 D).

## Results

In our case, a multitier architecture design was used with the process model described above. This process model contained a detailed definition of interrelated processes described in a way that would be understandable to the lay reader. The goal was a complete description of all of the events that take place in the organization (in our case at the Ministry of Health). In many cases, the current state (as it is) is described at the beginning, and only then is the future state (future) defined. As the procedural description of the drug record was up to date, it was decided to immediately describe the future state of vaccination against COVID-19 and thus describe the necessary procedural and legislative changes.

At the beginning of our work, we had various meetings with participants. From the written substantive intent, we continued through initial meetings with legislation experts from the Ministry of Health. Using knowledge gained from these initial meetings, we conducted interviews with pharmacy associations, patient organizations, and representatives of health insurance companies. Finally, with the basic process model, we arranged team workshops to define what would change, and these were directly incorporated into the already existing procedural architecture. In many cases, the requirements were written verbally, and only then was the process architecture created from this description. In commercial projects, this step is known as the “business architecture” (which is also often referred to as the operational or process architecture). Given that the EPS project was implemented by the government, we decided to use the term “process architecture” in this case. Already at this stage, the first inconsistencies began to appear. Therefore, some clarifications in the transcript were needed from the verbal—and less accurate—description of the process (precisely defined), and the process architecture itself resolved some inconsistencies present in its first version. All of these clarifications and decisions were made in consultations with the contracting authority at the Ministry of Health.

The specialty of the presented project was based on the very essence of the state-guaranteed system. Legislative rules define every detail of any health information system (HIS) used. The related legislation must therefore be flawless and must be closely linked to the proposed procedural model of the HIS under consideration. The roles and responsibilities of each entity must also be clearly defined. If the design is defective, some parts of the intended service may not be possible or, in the case of a mandatory system, entities may not be required to use it.

Many people often draw pictures as part of their discussions and there are many flip charts or similar devices available to support such discussions; far too many PowerPoint presentations are generated as a consequence. The method that we decided to use provides all parties with an analytic tool comprising visualizations. This is achieved by enabling drawing more informative pictures that can enhance any discussion. The process model is especially helpful in cases of unclear processes in an environment characterized by a high frequency of changes. Its clarity allows lay people to orient themselves in the connections between processes and the connections between systems. At the same time, the process model enables a facilitated discussion between the various opinion groups involved in the process.

Animating the process via model simulation can then more clearly demonstrate both the mistakes in the model analysis and within the model being animated. It is useful to test the designs in advance of further process development since pretesting can save a lot of time and effort. In these cases, where the process does not work correctly, it is usually because a certain action was left out or an interface between people or system units was not defined correctly and properly. Mistakes could show dead-end paths, never-ending cycles, or incorrect cooperation between (or among) the process players.

Conventional wisdom says that one picture is worth a thousand words. However, based on our experience, we have concluded that animation of all possible process flows is worth 10-100 pictures. For one system that was developed, the documentation consisted of 400 pages of text, comprising 38,000 words. However, it was possible to represent the entire proposed system in a single animated picture. The process modeling software from CraftCASE [7] was used for the animation during the workshops and discussions. The resolved changes were recorded interactively. Subsequently, after the incorporation of changes and graphic fine-tuning, each version was sent (in PDF form) to all team members so that they could study it and prepare for the next meeting. Creating such a picture increased a common understanding of the problem; otherwise, a considerable degree of effort is required to identify the minuscule details of the system in the text document. Thus, a diagram can reveal details that were previously known.

The intention was to design the basic structure of processes and to define a complete process hierarchy above them. This approach enabled establishing a communication environment that was suitable not only due to its mathematical basis but also by providing an intuitive interface suitable for discussion with the professional public without the need for training or an explanation of methodology.

**Figure 2:**
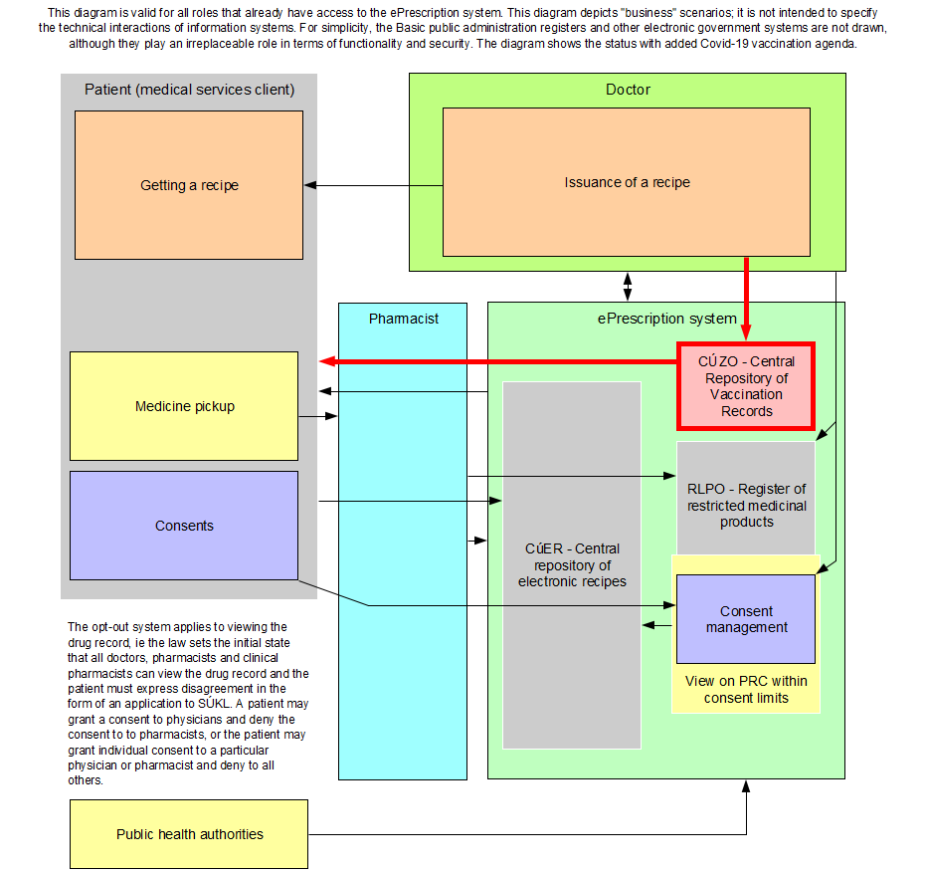
Final diagram of electronic prescription (ePrescription) process modules. SÚKL: State Institute for Drug Control.

Owing to the previous phase of the EPS model, sufficient knowledge of the issue and refined ideas were obtained. When this initial version of the main process modules was adapted to the legislation process, it had an astonishing effect: the diagram started to be the most highly demanded part of the legislation for many people who wanted to understand “how it works” quickly. The diagram provided a concise, simple, clear, and comprehensive view of what EPS issues are addressed, what is affected, who is involved, and how and which situations may arise. The final version of the diagram (as depicted in Figure 2) served as a unique discussion base for further development that was used for inclusion of the COVID-19 vaccination status into the EPS model. Participants’ ideas were gradually incorporated and subsequent versions were presented.

This final version of the diagram (also depicted in Figure 2, red square) was used as an annex to the explanatory memorandum to the draft amendment to the Pharmaceuticals Act introducing and describing the EPS/VR. This draft was accepted by the Czech legislative committee and came into effect on January 1, 2021.

## Discussion

Experience has shown that using conventional methods to draft new legislation for an issue as complex as the EPS for the inclusion of VR is a very lengthy process that normally takes 9-12 months, demanding a high number of discussions, meetings, and workshops. With the conventional method, a text with legislative intent is created immediately, and then the paragraph-by-section wording of the legislation is created and commented on directly. This process is then repeated over and over, as any change in any part of the text has to be checked and rechecked within the entire document. One of the main goals of the proposed approach was to significantly shorten this period. Therefore, we decided to use process modeling tools to create the EPS model.

Our method defines a formal architecture. First, process participants (eg, patients) are specified. Next, major groups of scenarios are added (eg, obtaining a prescription), followed by individual scenarios. Finally, connections between scenarios (arrows) are filled in. This creates a clear diagram that can be used for further discussion. For further explanation, the diagram can be enhanced with animation to enable better visualization of individual dependencies, time sequences, dead ends, conflicting interests, and other elements.

Before selecting our method based on the mathematical basis of state diagrams, we had tried other methods, but none of them provided a clear, widely understandable, and acceptable graphic notation. The unambiguity in connection with comprehensibility turned out to be the main advantage of our approach. This allowed speeding up the path to consensus among all parties involved and streamlined the direction of the discussion by resolving comments and conflicts more quickly.

Some authors have dealt with process modeling in health-related topics using the UML. However the UML diagram can be interpreted in several different ways, which may influence the overall result. Therefore, we decided to use modeling in the Business Object Relationship Modeling (BORM) methodology and state diagrams [6,8,9].

UML-based process models have previously been used in health care to depict an existing situation. One example involves the description of population analysis and proposed measures as a secondary method for outputting research on obstructive sleep apnea [10]. Another such example was for describing a specific method of caring for and treating pediatric asthma patients or to describe health care processes within a hospital [11]. A process model was also previously used to describe the current use of HL7 (as a set of international standards for the transfer of clinical and administrative data between different software applications) in the specific case of osteosarcoma [12]. We decided to use process modeling not only to depict the present state, but mainly to propose an entirely new process model for the complete discussion about the health system as the basis for defining the legislative model of the EPS.

New processes were previously proposed using modeling tools, such as for setting up standardized processes within breast cancer screening [13]. However, in that case, the model was used only to describe these processes, whereas we have used the process model as the entire working platform to reach a consensus among the various players within the health sector during preparation of legislation. We found that several rounds of sharing and improving the model were necessary.

Another process modeling project closely related to health care was in the context of social care to describe and standardize the process of solving the entrustment of children to the care of divided parents. The authors affirmed that custody of children was optimized and accelerated by the active use of process models [14]. Nevertheless, these process diagrams were never used as an annex to the legislation, as in our case.

BMPN is another process modeling method that is a graphical notation used to illustrate the steps in a business process. BMPN was used to verify General Data Protection Regulation compliance in the wider health care area [15]. However, as above, the process modeling was only used to describe existing legislation, whereas we have used this approach to define the fundaments of a legislative norm and to reach consensus. BPMN has also been applied in the context of task planning to identify, describe, and find typical process patterns and scenarios [5]. Using BPMN as part of openEHR—which is an open standard specification in health informatics that describes the management and storage, retrieval, and exchange of health data in electronic health records—can improve understanding of that approach using the process visualization. Although this approach focuses on modeling existing scenarios, we adapted the use of process modeling during discussions and definitions as the main method during the creation phase of legislation and scenarios creation.

Process visualization is also commonly used in the creation of journey maps [16]; however, in this case, it is focused on the point of view of a single person (participant) that can be compared to journey maps of other team members. By contrast, our method provides a complex view of the entire process across all participants.

Complex causal process diagrams [17] have also been used in a health care context for specifying the causal linkages in complex systems, organizing interdisciplinary research, and serving as a basis for modeling. These diagrams offer a transparent way of making complex processes understandable to the wider public. However, our procedure allows the process diagrams to be used for other purposes as well. For example, the diagrams can be used to validate that there are no elements missing or to simulate the course of the decision-making process. Moreover, one of the most important features of our procedures is the ability to explain the changes that emerged during the discussion by using an animated process.

A structural equation model diagram of eHealth implementation issues in low-resource countries was used to visualize the results of research surveys [18]. Although this approach was used to compare results across selected countries, a process model was not used to define categories or even the subjects of research. There are several other related methods and approaches that have been systematically reviewed elsewhere [19].

Our approach is unique by using hierarchical state machines–based process modeling during the process of health care legislation creation. When the EPS was introduced in 2008 using the classical legislative method, preparation of the legislation took over 1 year. When process modeling was used for the first time in 2019 to introduce the patient medication list in the EPS, it took 5 weeks to draft the whole bill and 3 months total from the first intent to the moment the bill was passed. In the case of VR incorporation in the EPS, the bill was drafted in 3 weeks and it took a total of 2 months to pass the bill. This makes a substantial difference, especially in times of crisis such as the COVID-19 pandemic. We believe that this approach is a novel contribution to the literature and can be widely adapted to other contexts, both within and outside health care, worldwide.

Nevertheless, the approach we used in this context may not be suitable for modeling processes with lower complexity. Direct discussions between stakeholders might suffice for such simple tasks, as process modeling would be too time-consuming and cumbersome. In addition, there are other very complex process areas that cannot be described by a single process model. In these cases, an overall diagram would lose a large amount of information and detail, which would make it difficult to understand. Comprehensibility for all users from different fields is the key to the success of the entire method. However, our method might be applicable to a very complex process if it is used repeatedly. In this case, process models would be divided into smaller parts and discussed separately, and then a large model would be constructed and discussed again.

In the future, we would like to focus on improving the methodology to solve even more complex issues than the incorporation of VR in an EPS. In the first step, we plan to find the key to dividing the complex process into individual interrelated parts. We would then apply the process model to simultaneously moderate the discussion within the individual parts and manage the requirements between the individual parts.

We have already used our method successfully in other areas (such as information technology systems or the definition of project assignments). In every case, we managed to reach an agreement with the involved interest groups quickly. Therefore, despite the above-mentioned limitations, we believe our method to be widely applicable to facilitate several users with different fields of knowledge in collaborating on and discussing a complex problem.

The process architecture model in the form of a graphic scheme has proven itself to be a valuable communication platform and facilitated negotiation between stakeholders, including regulatory authorities, as each party has different interests and each comes from a different background in terms of expertise.

In our case, the model was used for legislation when we needed to enhance the earlier defined model of the EPS with incorporation of the COVID-19 VR. This case represents only one successful example, but can be used directly to define and build an information system, as the process modeling tool can be used to describe a conceptual software model and capture its links to the processes and requirements of information system users. This approach helped us to eliminate various misunderstandings between eHealth experts and experts for the legislative process, which is the overwhelming cause of project failure. The key was to test and discuss the proposed process model before any further actions of parliamentary deliberations. The process architecture model is comprehensible for all users, including the medical community, information technology professionals, legislators, experts from practice, and other participants in the professional discussion.

Compared to conventional methods, we have shown that the process of modification of legislation in such a complex issue as the integration of COVID-19 vaccination status into an EPS model significantly simplifies the preparation of a legislative standard, while reducing the risk of misunderstandings, inaccuracies, and errors. This method also enables the authorities’ control over the whole process, as the process architecture model guarantees consistency, accuracy, and simplicity. Moreover, it allowed shortening the whole process from at least 1 year to several weeks, which enabled approval of our approach and ultimately led to the successful implementation of the COVID-19 VR in the legislative framework of the Czech Republic.

## Abbreviations

BORM: Business Object Relationship Modeling
BPMN: Business Process Model and Notation
EP: electronic prescription
EPS: electronic prescription system
HIS: health information system
HL7: Health Level 7
SÚKL: State Institute for Drug Control
UML: Unified Modeling Language
VR: vaccination record

## Data Availability

More details related to the main process modules of electronic prescriptions for the original state and with added vaccination record shown in Figure 2A and Figure 2B, respectively, can be downloaded from the repository [20,21].
